# Identification of circular RNA hsa_circ_0044556 and its effect on the progression of colorectal cancer

**DOI:** 10.1186/s12935-020-01523-1

**Published:** 2020-09-01

**Authors:** Liang Jing, Junhui Wu, Xiaocheng Tang, Min Ma, Fei Long, Buning Tian, Changwei Lin

**Affiliations:** Department of Gastrointestinal Surgery, The Third Xiangya Hospital of Central South University, Changsha, Hunan China

**Keywords:** Noncoding RNA, Circular RNA, Colorectal cancer, hsa_circ_0044556, Biomarker

## Abstract

**Background:**

Circular RNAs (circRNAs) are a novel class of noncoding RNAs. Increasing evidence indicates that circRNAs play an important role in the occurrence and development of tumors. However, the role of circRNA hsa_circ_0044556 in the progression of colorectal cancer (CRC) remains unclear.

**Methods:**

First, we searched for differentially expressed circRNAs using a circRNA microarray in paired CRC and adjacent normal tissues. The circRNA hsa_circ_0044556 was screened out from the existing CRC circRNA microarray in the Gene Expression Omnibus database and our microarray. The clinical significance of hsa_circ_0044556 expression level in CRC patients was then investigated. Finally, the functions of the targets of this circRNA were determined in CRC cell lines.

**Results:**

Hsa_circ_0044556 was highly expressed in CRC patients and was positively correlated with tumor stage and lymph node metastasis. In CRC cell lines, the proliferation, migration, and invasion of cancer cells were inhibited by knocking down hsa_circ_0044556 expression.

**Conclusion:**

Hsa_circ_0044556 promoted the progression of CRC. It is possible that hsa_circ_0044556 will become a novel biomarker or therapeutic target for CRC.

## Background

Colorectal cancer (CRC) is a malignant cancer that seriously endangers the health of humans. Currently, the global incidence rate of CRC ranks third among cancer-related diseases with up to 1.2 million new cases each year, and more than 0.6 million deaths are expected each year [1]. From the perspective of the global incidence trend, the European and American regions are higher than the Asian and African regions, with the second place mortality rate (9.2%), and the developing countries are higher than the developed countries [2]. In 2015, China's cancer statistics showed that the incidence and the mortality of CRC ranked fifth among cancer-related diseases in China. The incidence of CRC and the death toll are rising yearly while the population with this disease trends younger [3]. Early diagnosis, accurate prognosis, and recurrence monitoring play important roles in cancer diagnosis and treatment. The 5-year survival rate of patients with advanced CRC is only 12%, while the 5-year survival rate of patients at the early stage can reach more than 90% [4]. Therefore, early diagnosis can significantly improve the prognosis as well as the survival and quality of life of patients with CRC. On this basis, the in-depth study of the pathophysiology mechanisms underlying the occurrence and development of CRC will help us more comprehensively understand this cancer and open up new ideas and methods for the diagnosis and treatment of CRC. However, accurate and reliable prognosis and recurrence monitoring methods for CRC patients are still lacking in clinical practice, which makes it impossible to perform detailed posttreatment management and recurrence monitoring for patients [5, 6].

Circular RNAs (circRNAs) are a new class of noncoding RNAs (ncRNAs), which are single-stranded circular RNAs with no free 5′-end cap or 3′-end poly (A) tail. They are produced by alternative splicing of a specific pre-mRNA (pre-mRNA). Most circRNAs consist of exons and may also contain intergenic or noncoding regions [7–10]. CircRNAs did not attract much attention from researchers at first. For a long time, circRNAs were considered by-products of incorrect alternative splicing. Then in 2012, Salzman et al. discovered a large number of circRNAs using high-throughput sequencing technology [7]. Now it is recognized that circRNAs are not as rare as previously thought. In contrast, circRNAs are highly stable in cells with high expression, and sometimes their expression levels are even 10 times higher than those of their homologous messenger RNAs. It has been found that circRNAs can act as a miRNA sponge bound to RNA-binding protein to exert biological functions.

In recent years, circRNAs have been considered to play an important role in tumor progression [11]. CircRNAs are expected to become biomarkers for tumor diagnosis and prognosis due to their stable circular structure [12]. Xu et al. found that hsa_circ_0001649 was expressed at abnormally low levels in intrahepatic cholangiocarcinoma tissues and could promote cell proliferation and tumor metastasis [13]. Circ-ITCH can act as a sponge of miR-7 and miR-20a to inhibit the negative regulatory effect of the latter on the target gene ITCH, while ITCH has a tumor suppressor function by inhibition of the Wnt/β-catenin signaling pathway [14]. However, the role of circRNA hsa_circ_0044556 in CRC remains unclear and deserves further investigation.

## Materials and methods

### Tissue collection

In this study, tissue samples of 52 patients with CRC from the Third Xiangya Hospital of Central South University, China between May 2018 and December 2018 were collected. No patient received radiotherapy or chemotherapy before surgery, and the postoperative pathological diagnosis was adenocarcinoma (high, medium, and low differentiation). For each specimen, two copies of cancer tissues and adjacent normal tissues were collected. The surgical specimens were cryopreserved in liquid nitrogen immediately after excision. This study was approved by the Ethics Committee of the hospital, and all patients gave informed consent.

### Cell culture

Human CRC cell lines (SW480, SW620, HCT116, HT29) and normal colonic epithelial cell lines (NCM460) were purchased from Wuhan Boster Biological Technology, Ltd. (Wuhan, China). SW480 and SW620 cells were cultured in L15 medium (Nanjing KeyGen Biotech Co., Ltd., Nanjing, China) containing 10% fetal bovine serum (FBS: Biological Industries Israel Beit-Haemek, Beit-Haemek, Israel). HCT116 and HT29 were cultured with McCoy's 5A medium (Nanjing KeyGen Biotech Co., Ltd.) containing 10% FBS. NCM460 was cultured in Dulbecco's Modified Eagle Medium (DMEM, Thermo Fisher Scientific™, Beijing China) containing 10% FBS. All cells were incubated at 37 °C in a 5% CO_2_ incubator.

### Cell transfection

siRNA-1, siRNA-2, siRNA-3, and negative-control siRNA were all designed by Suzhou Genepharma Co., Ltd., China. Cell transfection was done according to the manual of Lipofectamine 3000 (Invitrogen; Thermo Fisher Scientific, Inc.).

### Tablet cloning assay

The tumor cell lines that were successfully transfected with siRNA-3 were harvested, counted, and adjusted to a cell concentration of 500 cells/ml, then were seeded in 6-well plates (1 × 10^3^/well), and 3 duplicate wells were set for each cell line. The 6-well plates were incubated at 37 °C in a 5% CO_2_ incubator for 2 weeks. The cell culture medium was changed every 2 to 3 days. The 6-well plates were taken out after 2 weeks, and the medium was removed. Cells were then washed with phosphate-buffered saline (PBS) 2 times, dried naturally, and then fixed by adding 1 ml of 4% paraformaldehyde in each well. After cells were fixed at room temperature for 30 min, the paraformaldehyde solution was aspirated. Cells were dried and stained with 0.1% crystal violet for 30 min. After washing with PBS twice, the cells were photographed and counted.

### Cell Counting Kit-8 (CCK-8) assay

SW480 and HCT116 (5 × 10^3^/well) were mixed well in 100 µl normal medium containing 10% FBS, seeded into 96-well plates, and incubated at 37 °C in a 5% CO_2_ incubator. Each well received 10 μl of CCK-8 reagent at the specified time point (0, 24, 48, and 72 h). After incubation for another 3 h, the absorbance at 450 nm of each well was measured by an EnVision microplate reader (PerkinElmer, Inc., Waltham, MA, USA).

### Scratch test

A marker was used to draw horizontal lines with the help of a ruler on the back of a 6-well plate. The lines were evenly drawn to 1 cm long, and there were at least 5 lines for each well. A total of 5 × 10^5^ cells were inoculated into each well of a 6-well plate. Cells were transfected when they reached a density of approximately 70% confluence. When the cells grew to just cover the entire well, a 10 μl pipette tip was used to make a scratch, with the help of a ruler, perpendicular to the horizontal lines on the back of the plate. The detached cells were washed with PBS, and then serum-free medium was added to continue the culture for 72 h. Samples were taken at 0, 24, 48, and 72 h, the cells were photographed, and the scratch width was measured.

### Migration and invasion assays

Migration and invasion assays were performed using a transwell chamber (8 μm, 24-well insert; Corning Incorporated, Corning, NY, USA). After 3 × 10^5^ cells were mixed in 200 µl of serum-free medium, they were seeded in the upper chamber, 500 µl normal medium containing 20% FBS was added into the lower chamber, and then the plates were incubated for 48 h at 37 °C in a 5% CO_2_ incubator. The upper chamber and the lower chamber were washed twice with PBS. The cells in the upper chamber were cleaned with a cotton swab to remove cell debris. The chamber membrane was fixed with 4% paraformaldehyde for 30 min. The paraformaldehyde solution was aspirated and cells were dried, followed by 0.1% crystal violet stain for 30 min and two washes with PBS. Cells on the lower side of the chamber membrane were observed under an inverted fluorescence microscope (Olympus Corporation, Tokyo, Japan). The cells were photographed and cell numbers were calculated.

### Reverse transcription and real-time quantitative polymerase chain reaction (RT-qPCR)

Total RNAs in tissues and cells were extracted using TRIzol reagent (Invitrogen; Thermo Fisher Scientific, Inc. Waltham, MA, USA). RT-qPCR was performed according to the manual of the Toyobo RT kit (Toyobo Life Science, Osaka, Japan) and Hieff® qPCR SYBR® Green Master Mix (Yason Biotech Co., Ltd.). CircRNA primers were designed by Beijing Tsingke Biological Technology Co., Ltd. (Hunan, China). Glyceraldehyde-3-phosphate dehydrogenase (GAPDH) was used as the internal control, and the relative expression levels were calculated by the 2^−ΔΔCt^ method.

### CircRNA–miRNA–mRNA coexpression network construction

CircRNA–miRNA interactions were predicted using Arraystar’s homemade miRNA target prediction software (Rockville, MD, USA) based on TargetScan [15] and miRanda [16]. The miRNAs were scored and sequenced using the miRNA support vector regression (mirSVR) algorithm to focus on target miRNAs [17]. Therefore, for each circRNA, we identified the top 5 miRNAs in the miRVR score system to establish a top-5 circRNA–miRNA network (1 circRNA connecting to 5 miRNAs). To further predict the interactions between miRNA and mRNAs, the miRNA–mRNA overlapping set predicted by databases of miRDB [18], miRTarBase [19], and TargetScan were used to plot circRNA–miRNA–mRNA interaction networks in Cytoscape (version 3.4.0) [20].

### Gene Ontology (GO) and Kyoto Encyclopedia of Genes and Genomes (KEGG) enrichment analysis of circRNAs

The Database for Annotation, Visualization and Integrated Discovery (DAVID; https://david.ncifcrf.gov (Version6.7)) [21] is an online bioinformatics database that integrates biological data and analysis tools. It provides a complete set of gene and protein functional annotation information for users to extract biological information. KEGG is a database resource used to categorize high-level functions and biological systems from large-scale molecular datasets generated by high-throughput experimental techniques [22]. GO is an important bioinformatics tool for annotating genes and analyzing their biological processes [23]. To analyze the functions of circRNAs, the online database of DAVID was used for biological analysis. *P* < 0.05 indicated statistical significance.

### Data analysis: SPSS 19.0 software was used for statistical analysis (IBM Corp., Armonk, NY, USA)

The data were imaged using GraphPad Prism 6 software (GraphPad Software, Inc., La Jolla, CA, USA), and the data are expressed as the mean ± standard deviation. The differences between two groups were analyzed using the independent-sample t test, and intragroup differences were analyzed using one-way analysis of variance (ANOVA). *P* < 0.05 indicated that a difference was statistically significant.

## Results

### Expression profiles of circRNAs in human CRC tissues

**Fig. 1:**
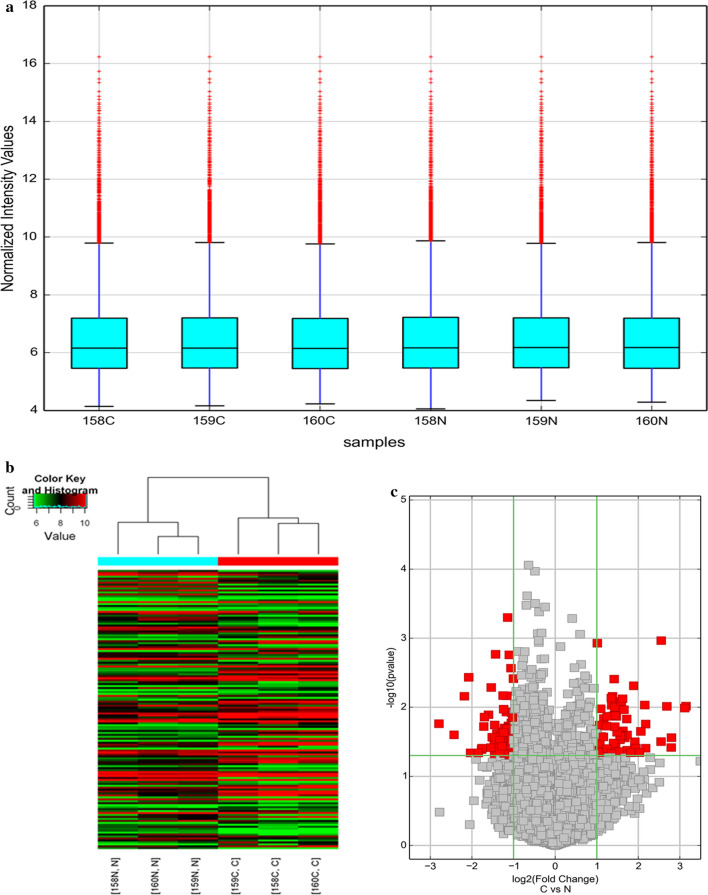
Differences in circRNA expression profiles between CRC and adjacent normal tissues. **a** Box plots show the distribution of circRNAs for the six samples (C for CRC and N for adjacent normal tissues). The distributions were nearly the same after normalization. **b** Unsupervised hierarchical clustering shows a distinguishable circRNA expression profiling among the six samples (C for CRC and N for adjacent normal tissues). Each column represents the expression profile of a tissues sample, and each row corresponds to a circRNA. “Red” indicates higher expression level and “green” indicated lower expression level. **c** Volcano plots showing differential expression of cicrRNA between the two groups. The red points represent the differentially expressed circRNAs with fold change ≥ 2.0 and P < 0.05

To study the expression profile of circRNAs in human CRC tissues, we used circRNA microarray technology to detect and analyze the expression of circRNAs in 3 pairs of CRC tissues and adjacent normal tissues. We drew a box plot showing the density distributions of all datasets after normalization and found that the distributions of log_2_ ratios were similar between all test samples (Fig. 1a). The unsupervised hierarchical clustering shows that circRNAs had different expression in CRC vs. adjacent normal tissues (Fig. 1b). The differential expression of these circRNAs was further confirmed in the volcano plot. With cutoffs of log_2_(fold change) ≥ 1 and *P* < 0.05 for differentially expressed circRNAs compared with adjacent normal tissues, 66 circRNAs in CRC tissues were upregulated, while 77 circRNAs were downregulated (Fig. 1c). Among the upregulated circRNAs, 52 were composed of exons, 9 were composed of introns, 1 was composed of intergenic regions, 2 were mixed circRNAs, and 2 were antisense circRNAs. Among the downregulated circRNAs, 67 were composed of exons, 4 were composed of introns, 1 was composed of intergenic regions, and 5 were mixed circRNAs (Additional file 1: Table S1).

### Identification of the target circRNAs in CRC

The circRNAs with upregulated expression in our microarrays (Additional file 1: Table S1) were compared with the circRNAs with upregulated expression in the CRC circRNA microarray GSE126094 [24] from the GEO database [25]. A total of 20 circRNAs were found to be upregulated in both microarrays, and the top 10 co-upregulated circRNAs are listed in Table 1. The top two circRNAs (hsa_circ_0004104 and hsa_circ_0044556) with upregulated expression in both microarrays were selected for further experiments. To further verify whether hsa_circ_0004104 and hsa_circ_0044556 had consistent expression between tissues and the microarray, we extracted total RNA from 10 pairs of CRC tissues and adjacent normal tissues for RT-qPCR validation. The results showed that compared with the adjacent normal tissue, hsa_circ_0044556 expression was upregulated in CRC tissues, which was consistent with the microarray results (Additional file 2: Figure S1A). The expression of hsa_circ_0004104 in CRC tissues and paracancerous tissues was not significantly different, which was inconsistent with the microarray results (Additional file 2: Figure S1B).

**Table 1:** The top 10 co-upregulated circRNAs in our microarrays and CRC circRNA microarray GSE126094 from the GEO database

CircRNA ID	Fold change	*P*-value	CircRNA type	Chromosome	Best transcript	GeneSymbol
hsa_circ_0004104	8.8323548	0.00959516	Exonic	chr5	NM_003118	SPARC
hsa_circ_0044556	6.9246896	0.02742728	Exonic	chr17	NM_000088	COL1A1
hsa_circ_0092283	4.5172247	0.03906942	Intronic	chr22	ENST00000216181	MYH9
hsa_circ_0004519	3.5141603	0.04530482	Exonic	chr16	NM_018124	RFWD3
hsa_circ_0028299	3.3084192	0.04170796	Exonic	chr12	NM_025247	ACAD10
hsa_circ_0000644	3.1460597	0.01080386	Intronic	chr15	ENST00000558261	RP11-351M8.1
hsa_circ_0004957	3.0425366	0.02561224	Exonic	chrX	NM_024917	TRMT2B
hsa_circ_0080425	3.0098437	0.00992264	Exonic	chr7	NM_022479	WBSCR17
hsa_circ_0070610	2.7063551	0.01320865	Exonic	chr4	NM_005443	PAPSS1
hsa_circ_0008365	2.6635341	0.0439912	Exonic	chr2	NM_006216	SERPINE2

### Expression characteristics of hsa_circ_0044556 in CRC

**Fig. 2:**
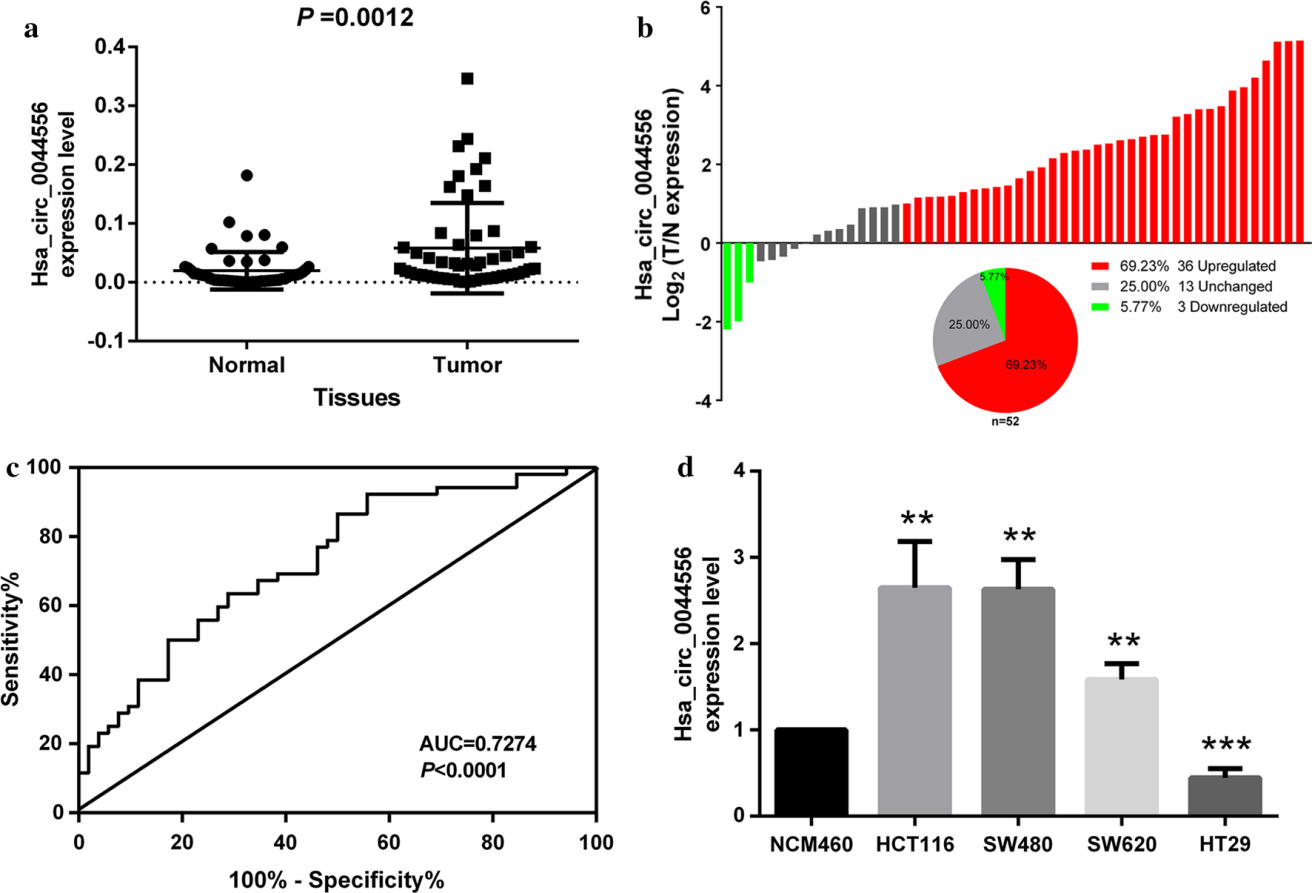
Has_circ_0044556 significantly upregulated in CRC tissues and cells had possibility as a novel biomarker for CRC. **a** The expression levels of has_circ_0044556 in the CRC tissues are significantly higher than those in adjacent normal tissues. **b** Histogram and pie chart of the proportion of CRC samples in which has_circ_0044556 expression was upregulated (36/52, 69.23%, red_, downregulated (3/52, 5.77%, green), or no change (13,25.00%, gray). Log2(T/N expression) value > 1 as significantly higher expression, which < − 1 as lower expression, and between **-**1 and 1 as no significant change. **c** Receiver operating characteristics (ROC) curve of has_circ_0044556 was built for differentiating CRC tissues from controls. The area under curve was 0.7274, P < 0.0001. **d** The expression levels of has_circ_0044556 in the CRC cell lines and NCM460

To further validate the clinical significance of hsa_circ_0044556, 42 pairs of CRC tissues and adjacent normal tissues were collected. We confirmed that hsa_circ_0044556 was highly expressed in CRC tissues compared to adjacent normal tissues (Fig. 2a) by RT-qPCR. Hsa_circ_0044556 was significantly upregulated in 69.23% (36/52) of CRC tissues compared to adjacent normal tissues (Fig. 2b). Based on the expression level of hsa_circ_0044556, the diagnostic value of hsa_circ_0044556 in distinguishing CRC from adjacent normal tissues was calculated using the receiver operating characteristic (ROC) curve. The area under the ROC curve (AUC) was 0.7274 (*P* < 0.0001) (Fig. 2c).

To further investigate whether the high expression level of hsa_circ_0044556 in patients was related to clinicopathological parameters, Table 2 was developed. We can see from Table 2 that the expression level of hsa_circ_0044556 did not have a significant difference in terms of patient sex (*P* = 0.3548), age (*P* = 0.562), tumor size (*P* = 0.9865), or position (*P* = 0.8677), but a significant difference was observed for tumor stage (*P* = 0.0121) and lymph node metastasis (*P* = 0.0045) (Table 2).

**Table 2:** The Associations between the hsa_circ_0014130 expression level and clinicopathological characteristics of patients with CRC

**Characteristics**	**No. of patients (%) **	**Hsa_circ_0044556 (Mean ± SD)**	***P *value**
Gender			
Male	32 (61.54)	6.992 ± 1.614	0.3548
Female	20 (38.46)	4.934 ± 1.029	
Age(years)			
≥ 60	24 (46.15)	5.521 ± 1.596	0.562
< 60	28 (53.85)	6.783 ± 1.461	
Tumor diameter			
≥ 4.5	28 (53.85)	6.217 ± 1.477	0.9865
< 4.5	24 (46.15)	6.180 ± 1.587	
Tumor site			
Colon	25(48.08)	6.388 ± 1.699	0.8677
Rectum	27(51.92)	6.026 ± 1.364	
Tumor stage			
I–II	23 (44.23)	3.233 ± 0.6187nn	0.0121*
III–IV	29 (55.77)	8.553 ± 1.749	
Lymphatic metastasis			
Yes	27 (51.92)	9.054 ± 1.843	0.0045*

*P < 0.05, compared among different groups. The expression level of hsa_circ_0044556 was significantly associated with tumor stage and lymphatic metastasis

Next, we used CRC cell lines for experiments. Compared to in the colonic epithelial cell line NCM460, hsa_circ_0044556 was highly expressed in the CRC cell lines of HCT116, SW480 and SW620 but was lower expressed in the CRC cell line HT29 (Fig. 2d). Because hsa_circ_0044556 was expressed at a relatively higher level in the two cell lines HCT116 and SW480, these two cell lines were chosen for further experiments.

### Loop-forming validation and siRNA design of hsa_circ_0044556

**Fig. 3:**
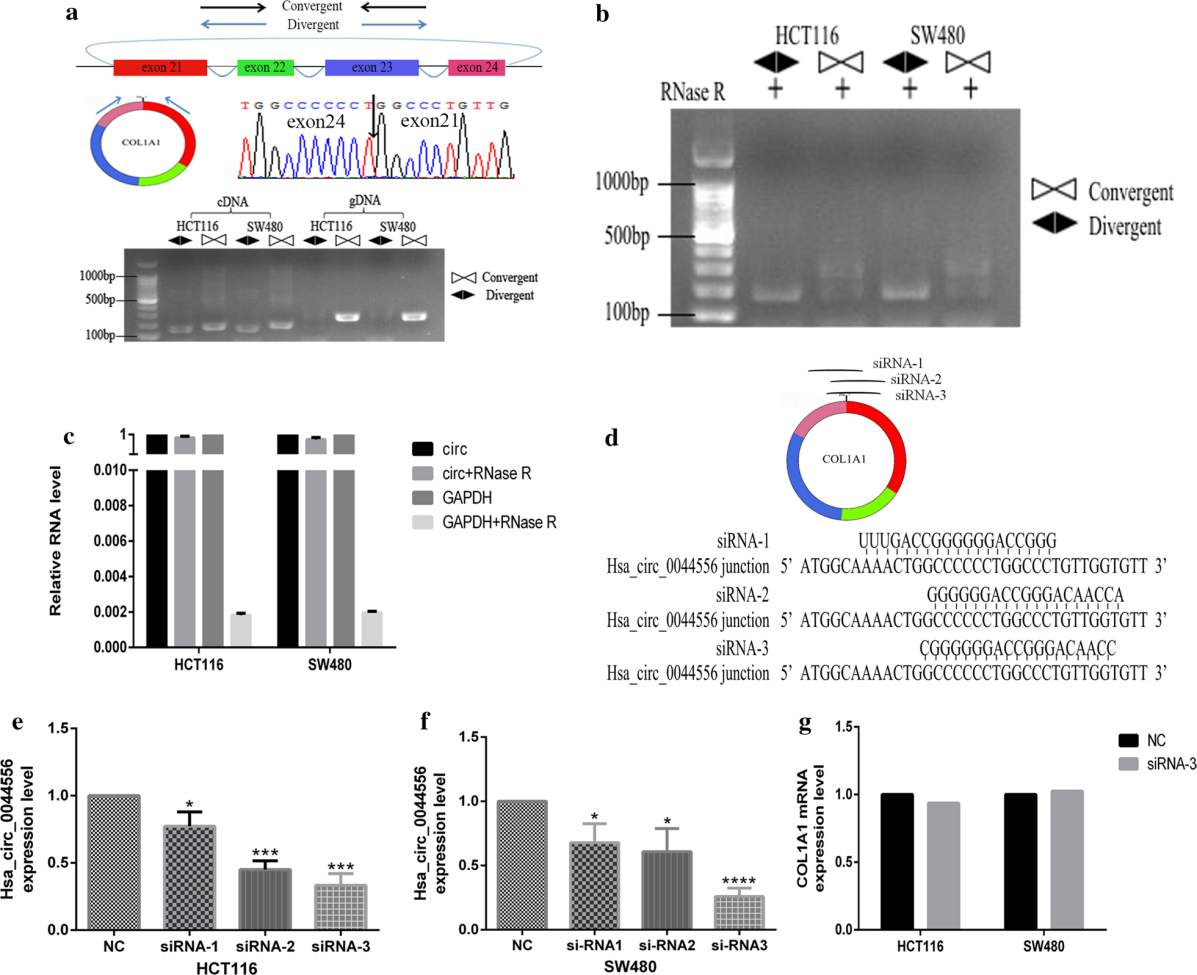
Has_circ_0044556 is a circRNA. **a** Verification that has_circ_0044556 is a circRNA, using divergent and convergent primers. Top, schematic illustration of has_circ_0044556 locus with specific primers. Bottom, RT-PCR products with divergent primers showing circularization of has_circ_0044556 gDNA, genomic DNA. Middle, Sanger sequencing result of has_circ_0044556. **b** RT-PCR products with Rnase R treatment showing circularization of has_circ_0044556. **c** The expression levels of has_circ_0044556 and GAPDH in the presence or absence of Rnase R treatment, respectively. **d** schematic illustration showed three targeted siRNAs. SiRNA targets the back-splice junction of has_circ_0044556. **e**, **f** qRT-PCR for has_circ_0044556 in CRC cells treated with three siRNAs. **g** The expression levels of COL1A1 mRNA treated with siRNA-3

By reviewing the human reference genome, we found that hsa_circ_0044556 (chr17: 48271490–48272189) was composed of exons 21–24 of the collagen type Ι alpha Ι (*COL1A1*) gene and was located on human chromosome 17q21.33. By RT-qPCR, Sanger sequencing, and anti-ribonuclease R digestion in HCT116 and SW480 cells, we verified that hsa_circ_0044556 had a circular structure (Fig. 3a–c). To evaluate the biological effects of hsa_circ_0044556 in CRC cells, we constructed three siRNAs covering the back-splicing region of hsa_circ_0044556. The results showed that the expression level of hsa_circ_0044556 in CRC cells transfected with siRNA-3 significantly decreased, while *COL1A1* mRNA expression level did not change significantly (Fig. 3d–g). Therefore, hsa_circ_0044556 was chosen for further experiments.

### Silencing hsa_circ_0044556 inhibits the proliferation, migration, and invasion of CRC cells

**Fig. 4:**
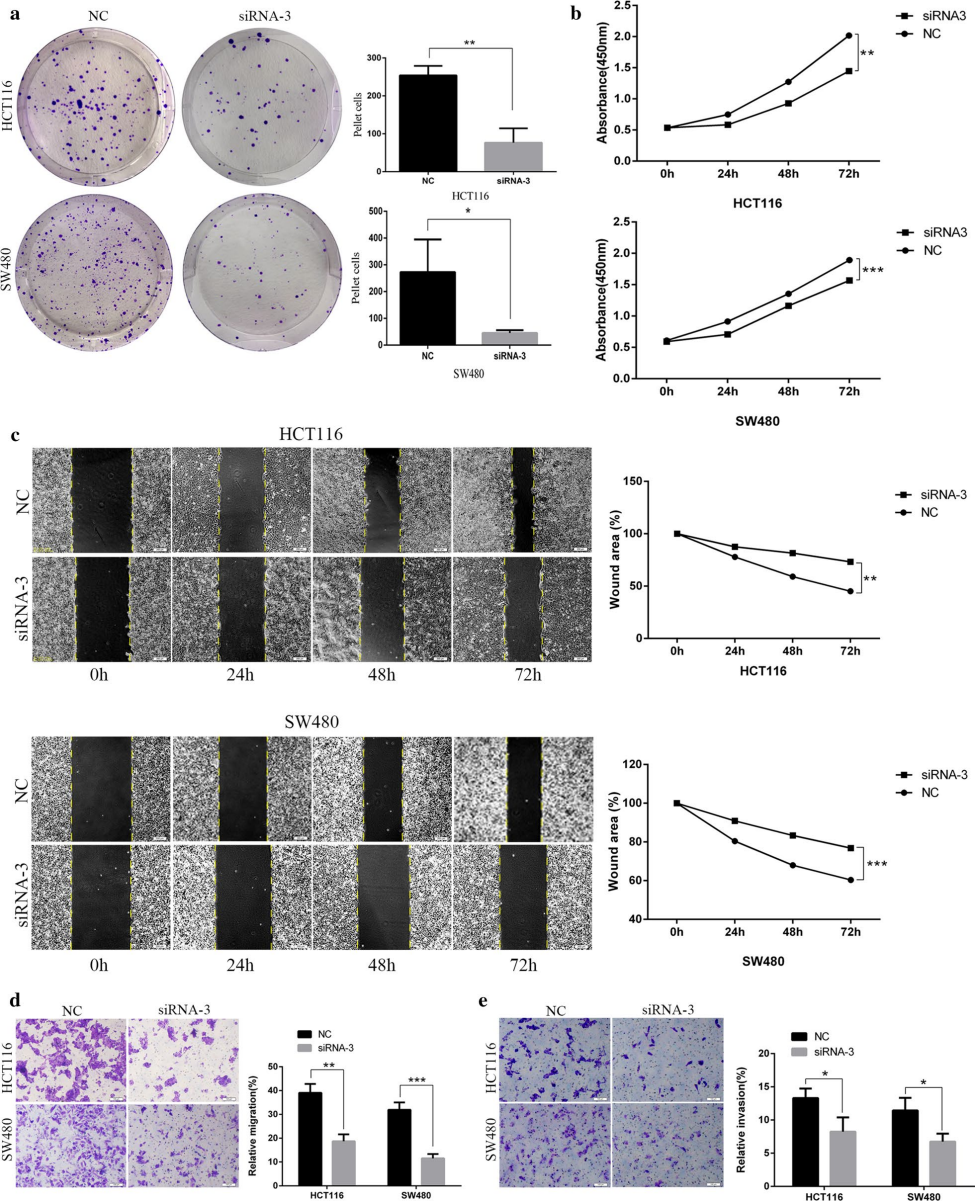
Silencing has_circ_0044556 inhibited proliferation, migration and invasion. **a**, **b** Silencing has_circ_0044556 decreased cell proliferation capacity. **c**–**e** Has_circ_0044556 knockdown suppressed migration and invasion of CRC cells. (*P < 0.05, **P < 0.01, ***P < 0.001

The results of the tablet cloning assay and CCK-8 assay showed that silencing of hsa_circ_0044556 lowered the proliferation ability of HCT116 and SW480 cells (Fig. 4a, b). The results of the scratch test and transwell experiment showed that knockdown of hsa_circ_0044556 inhibited the migration and invasion capability of HCT116 and SW480 cells (Fig. 4c–e).

### CircRNA–miRNA–mRNA coexpression network of hsa_circ_0044556

**Fig. 5:**
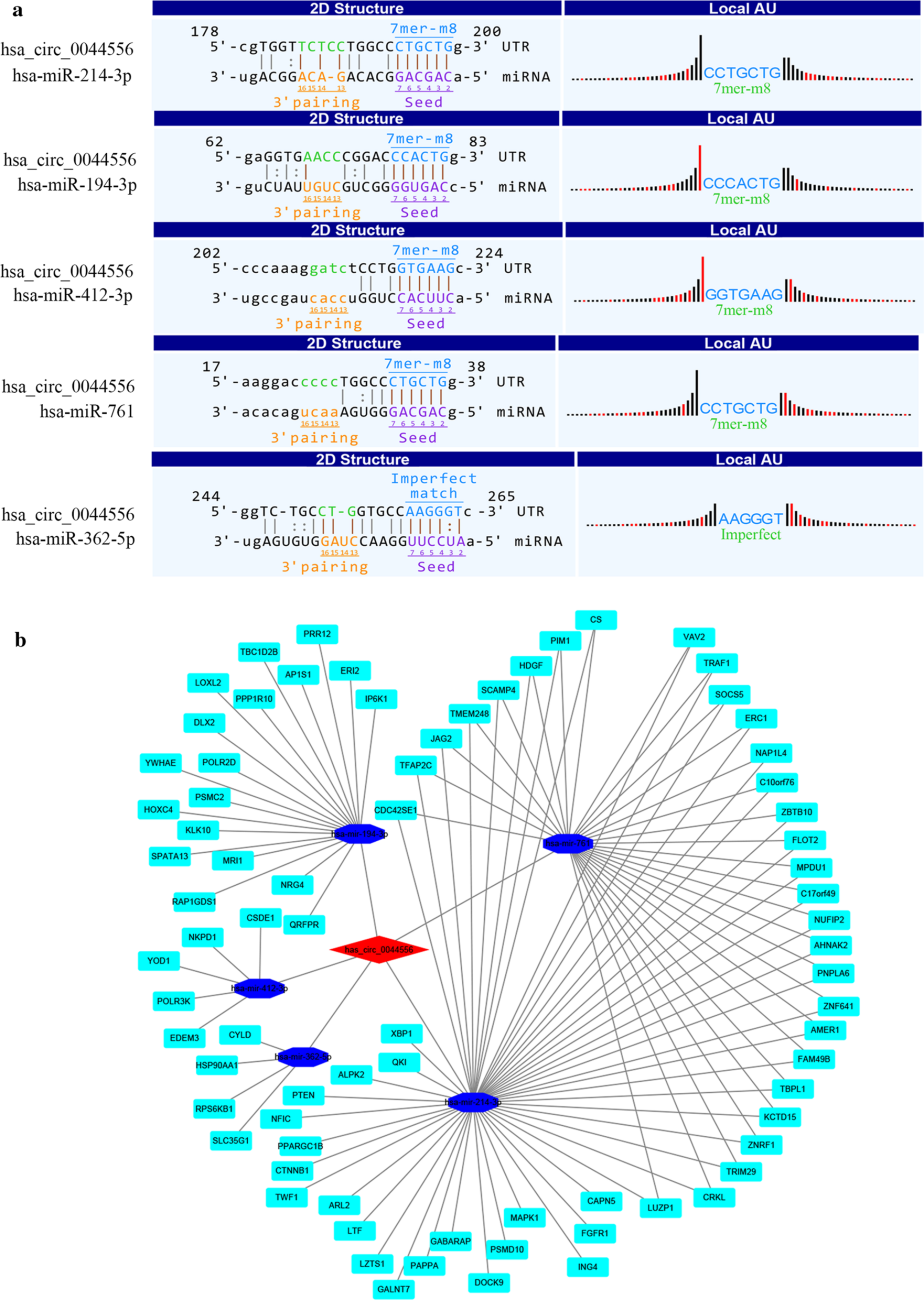
Bioinformatics prediction of has_circ_0044556 in CRC. **a** The five highest-ranking candidate miRNAs matched has_circ_0044556. **b** The co-expression network was drawn with the cytoscape software. Five miRNAs and their mRNA target genes were found with overlapping results

We hypothesized that hsa_circ_0044556 acts as a miRNA “sponge” to regulate circRNA–miRNA–mRNA networks. Using miRNA target prediction software, five miRNAs with the highest miRVR scores that might be bound by differentially expressed circRNAs were identified by TargetScan and miRanda (Additional file 1: Table S1). The molecular interaction between hsa_circ_0044556 and the 5 miRNA targets is depicted in Fig. 5a. Then, miRDB, miRTarBase, and TargetScan were used to predict the target genes that those five miRNAs might bind to. A total of 107 predicted genes (Additional file 3: Table S2) from the 3 databases were selected as the potential target genes of hsa_circ_0044556. Cytoscape analysis of the circRNA–miRNA–mRNA interaction network of hsa_circ_0044556 revealed that hsa-mir-214-3p and hsa-mir-761 exhibited the most complex interaction network, followed by hsa-mir-194-3p, hsa-mir-412-3p, and hsa-mir-362-5p (Fig. 5b). Since predicted target miRNAs only hsa-mir-214-3p proved to be downregulated in cancer progression in both ENCORI [26] and UALCAN [27] (Additional file 4: Figure S2A, B and C). Through further verification, we found that down-regulation of the expression level of hsa_circ_0044556 in HCT116 and SW480 cells increased the expression level of hsa-mir-214-3p (Additional file 4: Figure S2D).

### Bioinformatic analysis of the predicted genes in hsa_circ_0044556

**Fig. 6:**
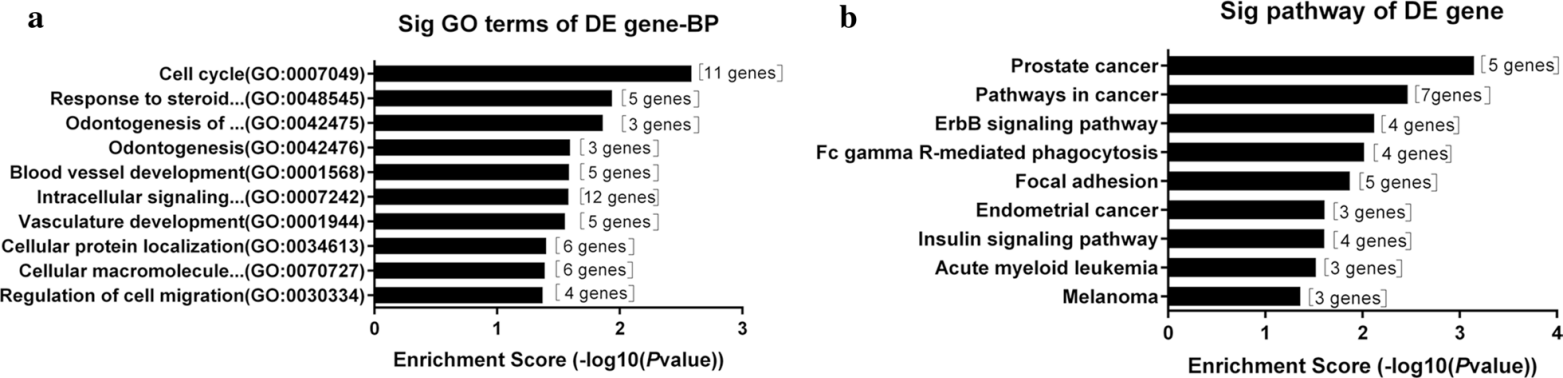
Gene ontology (GO) enrichment analysis and KEGG pathway analysis for has_circ_0044556. **a** The top 10 significantly enriched target genes and their scores are listed as the x-axis and the y-axis, respectively. **b** The top 9 significantly enriched pathways and their scores were listed as the x-axis and the y-axis, respectively

Gene ontology (GO) analysis was performed on hsa_circ_0044556, and the functional roles of the top 10 most enriched target genes were investigated from the perspective of biological processes (Fig. 6a). The results showed that hsa_circ_0044556 had a strong correlation with the cell cycle, responses to steroid hormone stimulation, and angiogenesis. KEGG analysis of hsa_circ_0044556 indicated that its top nine enriched pathways included prostate cancer, the cancer pathway, and the ErbB signaling pathway (Fig. 6b). Seven target genes were enriched in the cancer pathway. These data suggested that hsa_circ_0044556 may play important roles in the malignant behavior of cancer by regulating the expression of target genes involved in these pathways.

## Discussion

In recent years, with the rapid development and extensive application of RNA sequencing technology, circRNAs have become a hotspot in the field of RNA research. Researchers have found that many exon transcripts can form circRNAs through nonlinear reverse splicing or gene rearrangement. Moreover, they account for a large percentage of all spliced transcripts [7]. CircRNAs may come from introns or exons [28]. In mammals, exon-constituted circRNAs have two mechanisms of loop formation: lariat-driven circularization and intron-pairing-driven circularization [7, 29, 30]. A covalently closed circular structure without a 5′-to-3′ polarity or poly(A) tail is then formed by reverse splicing of a typical splice.

More and more studies have investigated the potential functions of circRNAs in various diseases, such as nervous system diseases, cardiovascular diseases, and cancers [31–34]. Some abnormally expressed circRNAs have been associated with the tumor development, invasion, metastasis, or prognosis of patients [35–41]. In mammalian cells, compared with other ncRNAs, such as miRNAs and long noncoding RNAs, circRNAs have highly conserved sequences and high stability [42]. These features might let circRNAs become ideal biomarkers and potential therapeutic targets for disease diagnosis.

In this study, high-throughput circRNA microarrays were used to study the expression of circRNAs in human CRC. The results showed that the expression of circRNAs in CRC tissues (n = 3) was significantly different from that in adjacent normal tissues (n = 3) (Fig. 1). Compared with the adjacent normal tissues, our microarray data showed that 66 circRNAs were significantly upregulated while 77 circRNAs were significantly downregulated in CRC tissues (Additional file 1: Table S1). We verified the expression levels of two circRNAs (hsa_circ_0004104 and hsa_circ_0044556) in 10 pairs of CRC and adjacent normal tissue samples, and only hsa_circ_0044556 was determined to be upregulated in CRC tissues, and with statistical significance (*P* = 0.0304) (S Additional file 2: Figure S1A). The expression level of hsa_circ_0004104 was inconsistent with the microarray results, and there was no statistical significance (*P* = 0.9208) (Additional file 2: Figure S1B). The above results indicate that validation of differently expressed circRNAs in microarray analysis is an important step in such a screening study. In addition, to verify the results above, we expanded the sample size to detect the expression of hsa_circ_0044556 in other tissue samples (n = 52). The results showed that hsa_circ_0044556 was significantly upregulated in 69.23% (36/52) of CRC tissues, with an average increase of 6.65-fold compared to the adjacent normal tissues. ROC analysis showed that hsa_circ_0044556 level had relatively high sensitivity and specificity, with an AUC of 0.7274 (Fig. 2). More importantly, considering the clinical pathological factors, we found that the high expression level of hsa_circ_0044556 in CRC was closely related to tumor stage and lymph node metastasis (Table 2), important factors in evaluating the prognosis of CRC. These results indicate that hsa_circ_0044556 might be involved in the progression and metastasis of CRC and could be used as a potential biomarker and a new therapeutic target for CRC.

One of the most important things about circRNAs is that they act as miRNA sponges. Certain specific circRNAs can bind and negatively regulate miRNAs involved in the competitive endogenous RNA (ceRNA) network, thereby regulating linear RNA transcription and protein production. Thomas et al. found that circRNA ciRS-7 can strongly inhibit miR-7 activity, leading to increased miR-7 target expression levels [40, 43]. Other functions may include gene expression regulation at the transcriptional or posttranscriptional level [44], and even encoding proteins [45, 46]. In this study, to further understand the biological functions of hsa_circ_0044556, 5 miRNAs with the highest mirSVR scores were identifed for each differentially expressed circRNA using miRNA target-prediction sofware (namely, hsa-mir-214-3p, hsa-mir-761, hsa-mir-194-3p, hsa-mir-412-3p, and hsa-mir-362-5p) (Additional file 1: Table S1). We used TargetScan, miRDB, and miRTarBase to predict the hsa_circ_0044556-miRNA–mRNA network. In essence, this network diagram shows a cellular RNA network with hsa_circ_0044556 interacting with 5 miRNA nodes and 107 target genes (Fig. 5b). Through the retrieval of ENCORI and UALCAN, it was found that hsa-mir-214-3p had low expression in colorectal adenocarcinoma. Furthermore, it have been found that hsa-mir-214-3p was significantly reduced in epithelial ovarian cancer cells and could affect epithelial ovarian cancer cell proliferation, invasion, increasing cisplatin chemosensitivity and inhibiting in vivo tumor growth proliferation by binding X-inactive specific transcript (XIST) [47]. It have also been reported that the downregulated hsa-miR-145-5p and hsa-mir-214-3p may modulate the expression of both EMT and NGAL/MMP-9 pathways [48]. In CRC lines, we also confirmed that silencing the expression level of hsa_circ_0044556 increased the expression level of hsa-mir-214-3p. Therefore, the decreased expression and inhibited function of hsa-mir-214-3p in CRC further support our hypothesis that hsa_circ_0044556 functions as a miRNA sponge to regulate the hsa_circ_0044556- hsa-mir-214-3p-mRNA network.

We found that a large number of mRNAs may participate in the above hsa_circ_0044556-miRNA–mRNA network, such as *ARL2*, *MAPK1*, and *PTEN*. Therefore, GO and KEGG pathway analysis was performed to detect the functions of these potential target genes. The results of GO enrichment analysis (Fig. 6a) showed that the target genes of hsa_circ_0044556 participated in the regulation of the cell cycle and angiogenesis, indicating that the regulation of these genes in the occurrence and development of CRC has importance in cell responses. The cancer pathway and the ErbB signaling pathway, two KEGG pathways correlated with hsa_circ_0044556 expression (Fig. 6b), may be related to the proliferation, migration, and invasion of CRC cells. Therefore, we speculate that the hsa_circ_0044556-miRNA–mRNA axis is a possible mechanism that promotes the development of CRC, and it is worthwhile to further study the overexpression of hsa_circ_0044556 as an inhibitor of miRNA and its possible mechanism of action.

In summary, this study revealed the expression profile of circRNAs in CRC tissues and demonstrated the abnormal expression of circRNAs in CRC. This study confirmed the significance of the upregulation of hsa_circ_0044556 and analyzed the relationship between hsa_circ_0044556 and the clinicopathological features of CRC patients, suggesting its potential role in the development and progression of CRC and its potential application as a CRC diagnostic biomarker. In the future, it will be necessary to explore the molecular mechanism of hsa_circ_0044556 as a miRNA sponge regulating the development and progression of CRC.

## Conclusion

Hsa_circ_0044556 promoted the progression of CRC. It is possible that hsa_circ_0044556 will become a new biomarker or therapeutic target for CRC.

## Supplementary information

**Additional file 1: Tables S1.** Results of circRNAs expression microarray in CRC.

**Additional file 2: Figure S1.** The expression levels of has_circ_0044556 and has_circ_0004104 in CRC were preliminarily verified.

**Additional file 3: Table S2.** Hsa_circ_0044556 common target genes in miRDB, miRTarBase and TargetScan.

**Additional file 4: Figure S2.** The expression levels of has-mir-214-3p in the CRC tissues and adjacent normal tissues.

## Data Availability

All datasets presented in this study are included in the article/additional files.

## Abbreviations

ARL2: ADP ribosylation factor-like protein 2
ANOVA: Analysis of Variance
cDNA: Complementary DNA
CEA: Carcinoembryonic antigen
circRNAs: Circular RNAs
COL1A1: Collagen type 1 alpha 1
CRC: Colorectal cancer
DAVID: The Database for Annotation Visualization and Integrated Discovery
DMEM: Dulbecco's Modified Eagle Medium
DNA: Deoxyribonucleic acid
ENCORI: The Encyclopedia of RNA Interactomes
FBS: Fetal bovine serum
GAPDH: Glyceraldehyde phosphate dehydrogenase
GEO: Gene expression omnibus
GO: Gene Ontology
ITCH: Itchy E3 ubiquitin protein ligase
KEGG: Kyoto Encyclopedia of Genes and Genomes
MAPK1: Mitogen activated protein kinase 1
miRNA: MicroRNA
mRNA: Messenger RNA
NC: Negative control
ncRNAs: Noncoding RNAs
OB: Occult blood
ROC: Receiver operating characteristic
PBS: Phosphate buffered saline
PCR: Polymerase chain reaction
PTEN: Phosphatase and tensin homolog
RT-qPCR: Reverse transcription and real-time quantitative PCR
RNA: Ribonucleic acid
siRNA: Small interfering RNA
shRNA: Short hairpin RNA
SPSS: Statistical Product and Service Solutions
TCGA: The Cancer Genome Atlas
