# Supplementary figures and images for: Identification of circular RNA hsa_circ_0044556 and its effect on the progression of colorectal cancer
Source: Cancer Cell Int. 2020 Sep 1;20:427. doi: 10.1186/s12935-020-01523-1 (PMC7465356; doi:10.1186/s12935-020-01523-1)

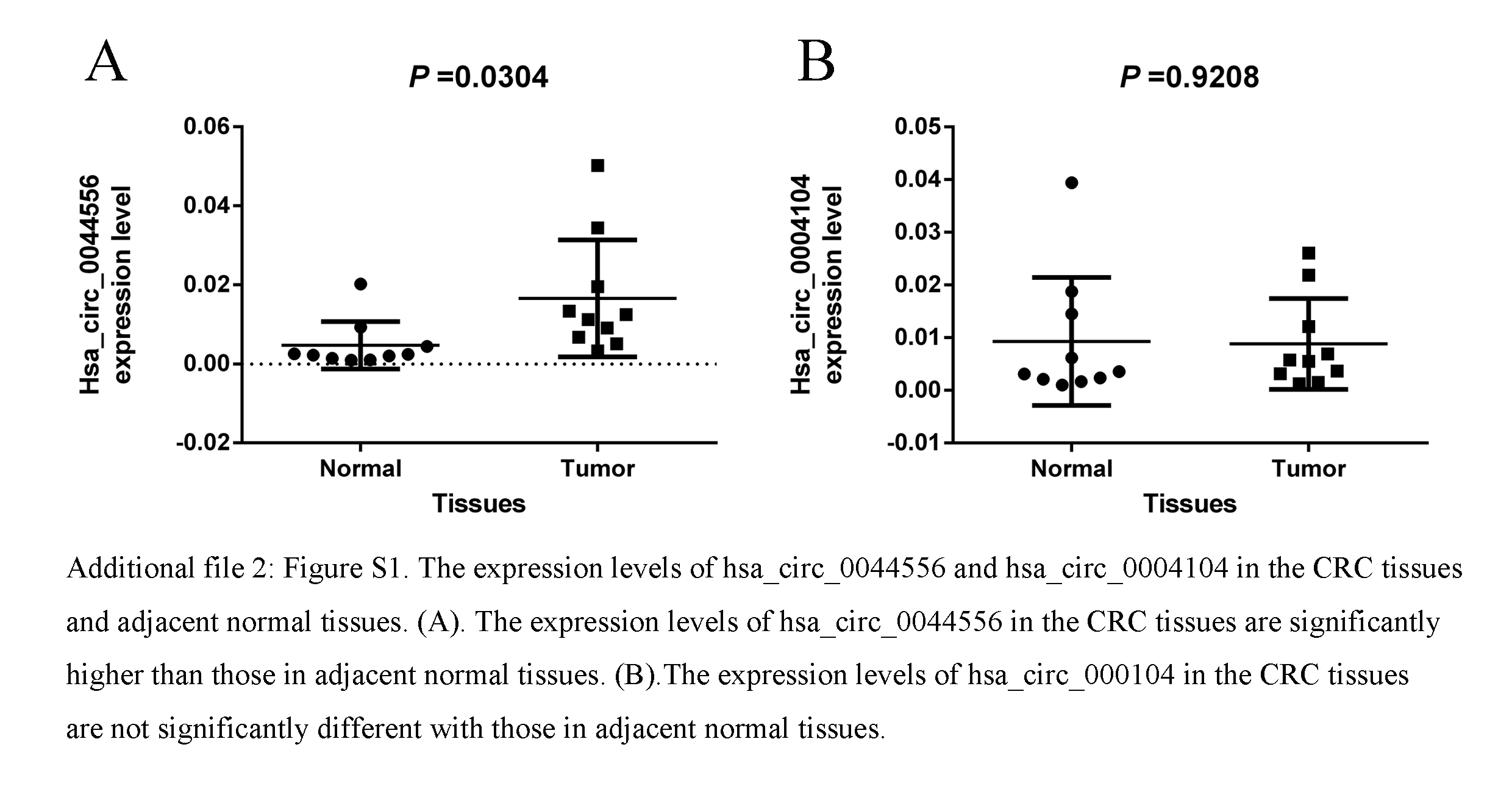

Supplement: Supplementary file 2 — Additional file 2: Figure S1. The expression levels of has_circ_0044556 and has_circ_0004104 in CRC were preliminarily verified. [file 12935_2020_1523_MOESM2_ESM.tif]

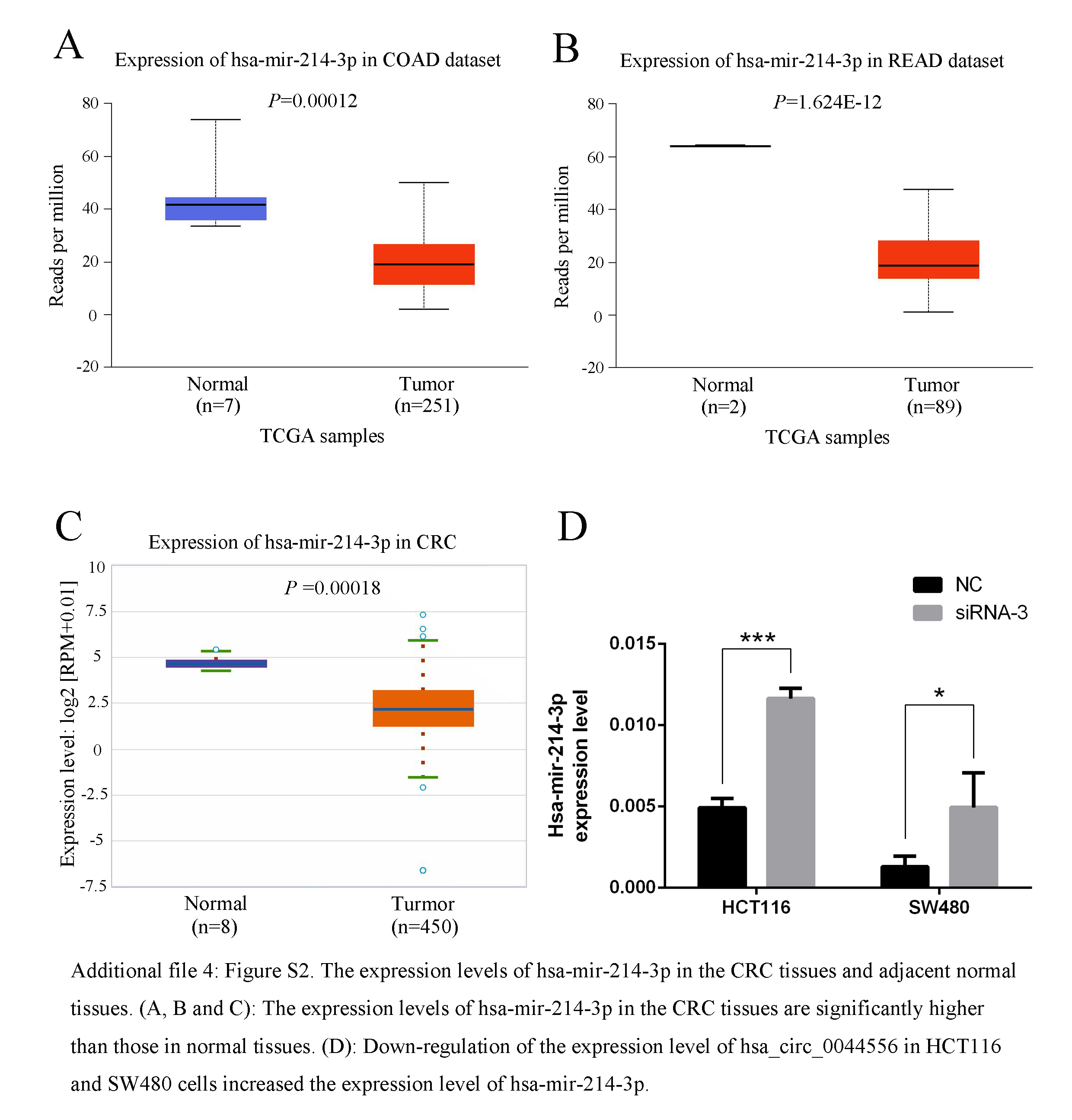

Supplement: Supplementary file 4 — Additional file 4: Figure S2. The expression levels of has-mir-214-3p in the CRC tissues and adjacent normal tissues. [file 12935_2020_1523_MOESM4_ESM.tif]
